# Treating HIV-associated cytomegalovirus retinitis with oral valganciclovir and intra-ocular ganciclovir by primary HIV clinicians in southern Myanmar: a retrospective analysis of routinely collected data

**DOI:** 10.1186/s12879-020-05579-2

**Published:** 2020-11-13

**Authors:** Jillian Murray, Adelene Hilbig, Theint Thida Soe, Win Le Shwe Sin Ei, Kyi Pyar Soe, Iza Ciglenecki

**Affiliations:** 1 Médecins Sans Frontières, Yangon, Myanmar; 2 Médecins Sans Frontières, Geneva, Switzerland

**Keywords:** Cytomegalovirus, Retinitis, HIV/AIDS, South-East Asia, Comorbidity, Valganciclovir

## Abstract

**Background:**

Cytomegalovirus retinitis (CMVR) is an opportunistic infection in HIV-infected people. Intraocular or intravenous ganciclovir was gold standard for treatment; however, oral valganciclovir replaced this in high-income countries. Low- and middle-income countries (LMIC) frequently use intraocular injection of ganciclovir (IOG) alone because of cost.

**Methods:**

Retrospective review of all HIV-positive patients with CMVR from February 2013 to April 2017 at a Médecins Sans Frontièrs HIV clinic in Myanmar. Treatment was classified as local (IOG) or systemic (valganciclovir, or valganciclovir and IOG). The primary outcome was change in visual acuity (VA) post-treatment. Mortality was a secondary outcome.

**Results:**

Fifty-three patients were included. Baseline VA was available for 103 (97%) patient eyes. Active CMVR was present in 72 (68%) eyes. Post-treatment, seven (13%) patients had improvement in VA, 30 (57%) had no change, and three (6%) deteriorated. Among patients receiving systemic therapy, four (12.5%) died, compared with five (24%) receiving local therapy (*p* = 0.19).

**Conclusions:**

Our results from the first introduction of valganciclovir for CMVR in LMIC show encouraging effectiveness and safety in patients with advanced HIV. We urge HIV programmes to include valganciclovir as an essential medicine, and to include CMVR screening and treatment in the package of advanced HIV care.

## Background

Cytomegalovirus (CMV) is a late-stage opportunistic infection (OI) in people living with HIV/AIDS, causing retinitis and extra-ocular end-organ disease, and is associated with increased mortality in HIV cohorts [1–3]. Retinitis is the most frequent manifestation of CMV; initial retinal scarring can directly affect the optic disc or fovea contributing to vision impairment, and progressive necrosis results in retinal detachment and blindness [1, 4, 5].

CMV retinitis (CMVR) was a common cause of blindness among patients with HIV/AIDS in the pre-antiretroviral therapy (ART) era [6]. CMVR is now uncommon in high-income countries (HIC) following universal introduction of ART. Although access to ART has also improved in resource-limited settings, the proportion of people presenting to care with advanced HIV disease (CD4 count < 200 cells/μL) remains high, and prognosis is poor [7]. The burden of CMVR in HIV cohorts in resource-limited settings is almost unquantified; a recent systematic review by Ford et.al (2013) showed high heterogeneity, with a pooled estimated prevalence in Asia of 14%, and a lower prevalence in Africa (2.2%) [8].

CMVR in resource-limited settings is a neglected disease, lacking access to screening, diagnosis and treatment [1]. Routine retinal examination is rarely performed. Therefore, the devastating reality of CMVR is that blindness is often the first indication of pathology owing to initial asymptomatic disease progression, and it is mostly irreversible [1, 9]. Although it has been shown that HIV clinicians can be trained to safely perform CMVR screening, diagnosis and treatment, this still requires education and access to specific equipment, including an indirect ophthalmoscope [10]. Largely, provision of such programmes remains limited to HIV clinics supported by non-government organizations (NGOs) [1].

Even when CMVR is suspected, treatment is rarely available in low- and middle-income countries (LMIC). Oral therapy, valganciclovir, replaced intravenous therapy as the standard of care for CMVR in HIC; however, LMIC continue to use local intraocular injection of ganciclovir (IOG) alone [1, 4, 5, 11, 12]. IOG is effective in stopping local CMVR progression and is affordable; however, it only treats the affected eye and does not prevent disease in the contralateral eye or treat systemic disease [1, 8, 13]. Additionally, intraocular administration presents an extra barrier: in many countries only ophthalmologists are trained to administer intraocular injections, and few of these specialists are available [10].

Despite proven feasibility of screening and improving access to appropriate systemic treatment, CMVR remains a neglected OI and is given little attention. We were unable to find any published record from LMIC demonstrating use of valganciclovir for CMVR. Here, we describe a small cohort of patients treated with valganciclovir in a Médecins Sans Frontières (MSF) supported HIV clinic in Dawei, Myanmar.

## Methods

### Setting

Dawei is a resource-limited port town in a rural division of southern Myanmar, bordering Thailand. Many residents temporarily migrate nationally and internationally for employment. In Myanmar, the HIV prevalence in the adult population is relatively low (0.7% in 2017) [14]; however, this predominantly consists of high-risk groups, including migrant workers, intravenous drug users, men who have sex with men, and commercial sex workers.

MSF has been providing comprehensive HIV services at Mittar Yeik (MTY) outpatient clinic in Dawei since 2004. MTY clinic offers HIV screening, HIV diagnosis and provision of ART (including second- and third-line ART), and standard screening and treatment of OIs. Clinical laboratory and pharmacy facilities are onsite. The active cohort of patients followed ranged between 2000 and 3000 in recent years, with between 130 and 300 new admissions per year, approximately half of which are patients with advanced HIV disease, with CD4 counts < 100 cells/μL (personal communication, routine programme data).

### CMV program

Since February 2013, diagnosis and treatment for CMVR has been provided at MTY. HIV clinicians with no previous ophthalmology experience were trained in indirect ophthalmoscopy and CMVR management, including administration of IOG, as described elsewhere [9, 10, 15, 16]. Indirect ophthalmoscopy was the only diagnostic strategy for CMVR available, as other investigations commonly utilized in HIC, including CMV polymerase chain reaction (PCR), were not available in rural southern Myanmar.

All patients with a CD4 count < 100 cells/μL, and any patients reporting ocular symptoms (blurred vision, floaters, scotoma, headache) irrespective of CD4 count, undergo eye screening on presentation at MTY. Patients with active CMVR are re-examined as clinically appropriate (weekly or biweekly) to monitor treatment response. Patients with cotton-wool spots are re-examined every 3 weeks until resolution, and those with a normal eye examination every 3 months until CD4 is > 100 cells/μL. Screening includes visual acuity (VA) testing using a three-metre Snellen chart in Burmese translation and complete retinal examination with an indirect ophthalmoscope through a fully dilated pupil (tropicamide 2 mg/0.4 mL, phenylephrine 5%).

Between February 2013 and February 2014, IOG was the only treatment available, administered as weekly intraocular injections of ganciclovir 2.5 mg until CMVR was inactive. Oral valganciclovir became available in February 2014. Patients with CMVR were treated with 900 mg of valganciclovir twice daily for 3 weeks, then 900 mg once daily until three conditions were met: retinitis completely inactive; CD4 > 100 cells/μl; at least 3 months of valganciclovir treatment completed.

After introduction of valganciclovir, IOG continued to be used in two situations. Firstly, if valganciclovir was contraindicated because of severe anaemia (haemoglobin < 8 g/dL), neuropathy or pregnancy. Secondly, to provide immediate local therapeutic drug levels in the vitreous in cases of CMVR deemed to be immediately vision threatening or extensive (over 25% of retinal surface).

Laboratory monitoring available included CD4 counts, renal function and haemoglobin. Follow-up haemoglobin levels were measures for patients in the systemic treatment group.

### Data collection and management

This study was a retrospective review of CMVR at MTY. All HIV-positive patients with a co-morbid diagnosis of CMVR from February 2013 to April 2017 were included. Patient baseline data was abstracted from MSF FUCHIA software (Epicentre, Paris, France). Additional clinical information was abstracted manually from charts. All data were entered into a Microsoft Excel database.

Patients were categorized with reference to the length of time they were on ART before CMVR diagnosis: “*new”* (ART less than 2 weeks, including naïve); *“on ART”* (ART more than 2 weeks); and “*interrupted treatment”* (including defaulters). Date of return to MTY was used as the initial date of visit for all defaulters. ART failure was defined as two consecutive viral loads of ≥1000 HIV RNA copies/mL [17]. Anaemia was diagnosed as haemoglobin < 13 g/dL for males and < 12 g/dL for females, whilst severe anaemia was defined as haemoglobin < 8 g/dL for both sexes [18].

Patient data included baseline VA and haemoglobin, treatment used (valganciclovir, IOG, both), treatment duration, VA on treatment cessation and CD4 trajectory. VA scores were translated into ICD-10 visual impairment scores for comparison (Appendix 1) [19].

The primary outcome was change in VA post-treatment, defined as an improvement or deterioration in VA in one or both eyes according to the ICD-10 classification. Mortality was analysed as a secondary outcome.

### Statistical analysis

Patients were stratified into two treatment groups for comparison: systemic (treatment including valganciclovir) and local (IOG alone) treatment. Categorical baseline and descriptive data were calculated as frequencies and proportions. Medians and inter-quartile ranges were calculated for continuous variables. Tests for differences between the groups were t-tests for continuous variables and Chi-squared or Fisher’s exact test, as appropriate, for categorical variables. Analyses were performed using R.

### Ethics

This manuscript is based on retrospective analysis of routinely collected data from long-standing project in Dawei, Myanmar. All the data was collected for purposes of patient’s follow-up and program monitoring. Confidentiality was respected, and no individual patient identifiers were revealed or used. As such, this analysis fulfilled exemption criteria of MSF ERB for retrospective use of routinely collected data as described in the manuscript, and it was exempted from formal ethics review.

## Results

### Baseline characteristics

Fifty-three patients with active CMVR were identified and included in analyses (Appendix 2). Median age was 37 (interquartile range [IQR] 34–42) years, and 29 (55%) were males (Table 1). Median CD4 at HIV programme entry was 18 (IQR 10–44) cells/μL.

**Table 1:** Baseline characteristics of HIV-positive patients with CMVR at MTY Clinic, Myanmar, 2013–2017

	Total (*N* = 53)	Local treatment (*N* = 21)	Systemic treatment (*N* = 32)	*P* value
Male sex, N (%)	29 (55)	10 (48)	19 (59)	0.576
Median age, years (IQR)	37 (34–42)	37 (34–42)	38 (34–46)	0.722
Year of CMV diagnoses, N (%)				< 0.001
2013	15 (28)	15 (71)	0 (0)	
2014	14 (26)	4 (19)	10 (31)	
2015	20 (38)	2 (9.5)	18 (56)	
2016	4 (7.5)	0 (0)	4 (13)	
Median (IQR) CD4 count, cells/μL^a^	18 (10–44)	31 (15–46)	16 (7.8–41)	0.103
Patient type, N (%)				0.472
New	31 (58)	13 (62)	18 (56)	
Interrupted treatment	3 (5.7)	2 (9.5)	1 (3.1)	
On ART	19 (36)	6 (29)	13 (41)	
Median (IQR) Hb, g/dL^b^	9.7 (9.1–11)	10 (9.2–11)	9.5 (8.1–10)	0.133
Bilateral retinitis, N (%)^c^	20 (38)	10 (48)	10 (31)	0.218

*ART* Antiretroviral therapy; *CMV* Cytomegalovirus; *CMVR* Cytomegalovirus retinitis; *Hb* Haemoglobin; *IQR* Interquartile range; *MTY* Mittar Yeik.

^a^ CD4 count within six months preceding CMVR diagnosis.

^b^ Before treatment.

^c^ Excludes one unknown.

Thirty-one (58%) patients were new to ART, 19 (36%) were on ART and three (5.7%) had treatment interrupted. Of those on ART, three (16%) had evidence of ART failure at the time of CMVR diagnosis. Forty-nine (92%) patients had anaemia at the time of CMVR diagnosis, with a cohort median haemoglobin before anti-CMV treatment of 9.7 (IQR 9.1–11) g/dL.

At programme enrolment, 20 (38%) patients had evidence of bilateral retinitis on indirect ophthalmoscopy, 32 (60%) had monocular retinitis, and baseline examination findings were unavailable for one (2%). A baseline VA was recorded for 103 (97%) of the 106 patient eyes screened. Of the 72 eyes with evidence of CMVR, 53 (74%) had mild/no visual impairment, nine (12.5%) had moderate impairment, nine (12.5%) were blind, and baseline acuity was missing for one (1%) eye.

### CMV therapy

All patients diagnosed with CMVR started anti-CMV therapy within 1 week of confirmation. Twenty-one patients received IOG alone. Most of these patients (*N* = 18) received treatment before valganciclovir was available at the clinic. Of the remaining three, valganciclovir was contraindicated due to anaemia in all patients, and two additionally had co-morbid nephropathy. Fourteen patients received valganciclovir alone, and the remaining 18 received both treatments.

Median duration of anti-CMV treatment was 98 (IQR 55–172) days (Table 2). Treatment duration was significantly longer in the systemic therapy group compared with the local treatment group (133 and 63 days respectively, *p* < 0.01).

**Table 2:** Primary and secondary outcomes for HIV-positive patients with CMVR treated at MTY Clinic, Myanmar, 2013–2017

	Total (*N* = 53)	Local treatment (*N* = 21)	Systemic treatment (*N* = 32)	*P* value
Median (IQR) duration of treatment, days^a^	98 (55–172)	63 (21–92)	133 (80–195)	< 0.01
Outcome, N (%)				0.665
Improvement	7 (13)	2 (10)	5 (16)	
Stable	30 (57)	11 (52)	19 (59)	
Deterioration	3 (6)	2 (10)	1 (3.1)	
Deceased	9 (17)	5 (24)	4 (12.5)	
Lost to follow up	1 (1.9)	0 (0)	1 (3.1)	
Unknown	3 (5.7)	1 (5)	2 (6.3)	
Worsening anaemia, N (%)	20 (38)	N/A	20 (63)	
CD4 count ≥100 cells/μL, N (%)^b^				
3 months	19 (36)	9 (43)	10 (31)	0.170
6 months	29 (55)	11 (52)	18 (56)	1
12 months	32 (60)	9 (43)	23 (72)	0.571

*CMVR* Cytomegalovirus retinitis; *IQR* Interquartile range; *MTY* Mittar Yeik; *N/A* Not applicable.

^a^ Excludes one patient treated with valganciclovir who was lost to follow-up.

^b^ Excludes nine patients who died (no CD4 count from follow-up).

At CMVR diagnosis, 33 (62%) patients were not taking ART. All 33 patients started ART a median of 14.5 (IQR 8.75–18) days following CMVR diagnosis.

### Changes in VA

Of the 72 eyes with active CMVR at the start of treatment, data were available for 57 (79%) at treatment completion (Table 3). Forty-four (61%) had mild/no impairment, six (8%) had moderate impairment, and seven (10%) were blind. No eyes developed new blindness during treatment. Eleven eyes belonged to patients who died, and four eyes had no VA recorded at treatment completion.

**Table 3:** Baseline and post-treatment VA by eyes affected for HIV-positive patients with CMVR treated at MTY Clinic, Myanmar, 2013–2017

	Total (*N* = 72)		Local treatment (*N* = 31)		Systemic treatment (*N* = 41)	
	Pre-Rx N (%)	Post-Rx N (%)	Pre-Rx N (%)	Post-Rx N (%)	Pre-Rx N (%)	Post-Rx N (%)
Mild/none	53 (74)	44 (61)	24 (77)	16 (52)	29 (71)	28 (68)
Moderate	9 (12.5)	6 (8)	2 (6)	3 (10)	7 (17)	3 (7)
Severe	0 (0)	0 (0)	0 (0)	0 (0)	0 (0)	0 (0)
Blind	9 (12.5)	7 (10)	5 (16)	5 (16)	4 (10)	2 (5)
Unknown	1 (1)	4 (6)	0 (0)	1 (3)	1 (2)	3 (7)
Deceased/LTFU	0 (0)	11 (15)	0 (0)	6 (19)	0 (0)	5 (12)

*CMVR* Cytomegalovirus retinitis; *LTFU* Lost to follow-up; *MTY* Mittar Yeik; *Rx* Treatment.

Considering VA outcomes by patient, following treatment completion, seven (13%) patients had improvement in VA, 30 (57%) had no change, and three (6%) had deterioration in vision (Table 2). One patient with bilateral disease deteriorated from bilateral mild/no visual impairment to bilateral moderate impairment, one patient with bilateral disease deteriorated from bilateral mild/no visual impairment to unilateral moderate impairment, and one patient with monocular disease deteriorated from mild/no impairment to moderate impairment. There was no difference in VA outcomes between treatment groups.

### Secondary outcomes

Nine (17%) patients died, with a median time from CMVR diagnosis to death of 51 (IQR 26, 86) days. Four (12·5%) patients receiving systemic therapy died, compared to five (24%) receiving local therapy (*p* = 0·19) (Table 2).

Twenty (62·5%) patients in the systemic therapy group experienced a decline from baseline haemoglobin at a median of 18 (IQR 11–29) days. A median haemoglobin nadir of 8·6 (IQR 6·93–9·68) g/dL was reached after a median of 24 (IQR 12·5, 45) days. Of these 20 patients, seven (22%) recorded an haemoglobin < 8 g/dL; two of this seven switched to IOG alone, three continued on VG, and two died prior to treatment change.

### CMV treatment cessation

Sequential repeat CD4 counts showed a sustained improvement in CD4 trajectory (Table 2). Reasons for anti-CMV treatment cessation are outlined in Table 4.

**Table 4:** Reasons for anti-CMVR treatment cessation in HIV-positive patients treated at MTY Clinic, Myanmar, 2013–2017

Reason for cessation, N (%)	Total (*N* = 44)^a^	Local treatment (*N* = 16)	Systemic treatment (*N* = 28)
CD4 > 100 cells/μL	20 (45)	3 (19)	17 (61)
No active retinitis	4 (9)	1 (6)	3 (11)
Unknown	18 (41)	10 (63)	8 (29)
Clinical decision re: blindness	2 (4.5)	2 (13)	0

*CMVR* Cytomegalovirus retinitis; *MTY* Mittar Yeik.

^a^ Excludes nine patients who died.

## Discussion

We describe the first experience of treating HIV-associated CMVR with valganciclovir in a LMIC and show encouraging treatment outcomes for patients receiving either a valganciclovir-containing regimen (alone or in combination with IOG) or local IOG treatment alone. All patients in our cohort received CMVR treatment, and all were either already on ART or started on ART within 14 days of CMVR diagnosis.

Our patients had a similar age and sex distribution to other CMVR cohorts [2, 15, 20]. The median CD4 count of 18 cells/μL was low, and lower than that reported from Yangon by Tun et al. (2011) (38 cells/μL) [15]. The high proportion of patients in Asia who present with low CD4 counts likely contributes to the large burden of CMVR disease in this region [8]. In our cohort, 74% of eyes with CMVR had mild/no visual impairment at diagnosis, consistent with the recognized phenomenon that most CMVR is asymptomatic at the time of diagnosis [1, 4, 21]. Such observations again support the need for universal screening for all patients with a CD4 < 100 cells/μL [1, 4]. After the inception of the CMVR programme in our clinic, the detection rate of CMVR among patients with CD4 < 100 cells/μL was 10.7% [16].

Most patients (58%) were new to ART at CMVR diagnosis. This reflects the protocol at MTY that newly diagnosed HIV-positive patients with CD4 < 100 cells/μL undergo immediate CMVR screening. The occurrence of CMVR in the ART-exposed group is likely explained by two factors. Firstly, immune reconstitution is delayed after ART is started, and CMVR can develop during this time [8, 21, 22]. Secondly, a proportion of ART-exposed patients were defaulters and those with treatment failure. Occurrence of CMVR in ART-exposed patients reinforces the need for clinically vigilant screening of all patients with CD4 < 100 cells/μL [4].

Visual acuity outcomes in our small cohort were encouraging. No patient developed blindness after treatment. VA improved in seven patients but deteriorated in three. Of the nine blind eyes at start of treatment, seven remained blind, one eye improved to mild/no vision impairment, and one eye belonged to a deceased patient. Blindness can occur as a complication of IOG administration; absence of new blindness supports previous evidence that primary HIV clinicians can be safely trained in this technique [15]. Repeated injections of IOG are associated with several potential vision-threatening complications, including endophthalmitis, vitreous haemorrhage and retinal detachment [1, 4, 8, 11, 13, 21, 23], but we did not record any of these in our cohort. Persistent blindness despite CMVR treatment is consistent with the often irreversible nature of CMVR-related vision loss, supporting the importance of early screening and treatment [1, 9]. The 11% of patients with vision loss in our cohort is lower than was found in the systematic review by Ford et al. (2008), which reported CMVR-related vision loss in 32% (range 0–75%) and bilateral loss in 28% of cases [8].

Baseline anaemia affected 92% of the cohort. Six patients had severe anaemia at baseline, who were, according to protocol, treated with IOG. Serial haemoglobin was only measured in patients receiving systemic therapy. Among 32 patients receiving valganciclovir, 20 (62.5%) experienced a decline in haemoglobin, with seven (22%) developing severe anaemia; two of these patients switched to IOG, three continued on valganciclovir (the reason for failure to switch per protocol was not documented), and two patients died prior to treatment change. Haemoglobin monitoring is a mandatory component of a CMVR treatment program, and reinforces the need for access to IOG as an alternative treatment option for this cohort.

The mortality rate in our cohort of patients with CMVR was 17%. The high mortality is consistent with the advanced HIV disease of our patients: the median CD4 count at baseline was 18 cells/μl, indicating profound immunosuppression. Previous data from MTY showed that approximately 53% of overall 10-year clinic mortality occurred within 12 months of ART initiation in the context of profound immunosuppression (WHO stage 3 or 4, 90.1%), consistent with literature supporting baseline CD4 count as an independent predictor of mortality [24]. In reviewing CMVR in LMIC, Ford et al. (2008) described a case fatality rate of 22%; however, no details such as CD4 count are provided, making comparison difficult [8].

Mortality was higher in the local treatment group (24%) than in the systemic group (12.5%). This should be interpreted with caution, as treatment was not randomly assigned; only IOG was available in the first year of the programme, while IOG alone was reserved for patients with contraindications to valganciclovir in later periods, and the number of events was small. Nevertheless, the finding is consistent with the literature; IOG targets local ocular disease, leaving patients at risk of retinitis in the untreated eye and systemic complications [1, 4, 21, 23, 25].

The need for systemic therapy is frequently overlooked, as diagnosis of non-ocular end-organ CMV disease is not possible in resource-limited settings, where biopsy, histology and PCR testing are often unavailable [8, 26]. Limited information on extra-ocular manifestations of CMV in LMIC exists; however, data from HICs indicate that disseminated CMV infection is present in 28.7% of HIV-positive patients [8, 26]. Autopsy data show CMV pathology to be present in 7–52% of HIV deaths in sub-Saharan Africa [27].

CMV viraemia and disease have been associated with increased risk of mortality in HIV cohorts, even in the ART era [2, 28]. There is an urgent need to improve CMV diagnosis in LMIC, which is limited to diagnosis of CMVR by indirect ophthalmoscopy, a technique that is rarely available. Detection of CMV viraemia by PCR is a promising option [29, 30].

IOG injections are invasive, are traumatic for patients and care providers, and are associated with possible complications including blindness. Although we and others have shown that HIV clinicians can be trained to safely perform IOG injections, this has not been scaled up [9, 10, 15, 16]. In many countries, only ophthalmologists are sanctioned to perform intraocular injections [1]. Despite advantages, valganciclovir is infrequently used in LMIC, with price considered a barrier [1]. IOG is cheap, costing US$0.57 per injection; however, this value does not account for other costs associated with this procedure, including the necessary equipment and trained personnel, and patient costs associated with travel and inability to work [1]. Valganciclovir is now included on the WHO Essential Medicines List, and is available at the reduced price of CHF3.33 for a 450 mg tablet [31]. For a standard three-month regimen (222 tablets), this would cost CHF739; approximately half our cohort completed treatment within 3 months. Valganciclovir is available at this price for ministries of health in LMIC and can also be accessed through Global Fund Requests [31]. We urge national programmes to access this life-saving drug.

Our analysis has several limitations. This was a retrospective analysis of routine data, with missing information often limiting analyses. Patients were not randomized to treatment groups, so our comparison of treatment outcomes is only descriptive. We have likely underestimated the burden of CMVR in our HIV cohort, as critically ill patients were transferred to hospital and may not have undergone eye screening. The possibility of a selection bias to more stable patients with less advanced CMVR exists, and outcomes should be interpreted with this in mind. Similar concerns have been echoed by other authors [2].

We were unable to assess for immune reconstitution uveitis (IRU), the well-recognized inflammatory condition arising from a reaction to CMV antigens that occurs as the immune system strengthens after ART is started, owing to a lack of specialized ophthalmology services [4, 21]. IRU causes blindness and visual deterioration. Whilst no new cases of blindness were identified, visual deterioration did occur in three patients; as we did not assess for IRU, it cannot be conclusively excluded. Finally, we were unable to reliably monitor for neutropenia and thrombocytopenia, recognized complications of valganciclovir treatment.

## Conclusion

We describe the first introduction of valganciclovir treatment for CMVR in LMIC, with encouraging effectiveness and safety in our cohort of patients with advanced HIV disease and profound immunosuppression. We urge HIV programmes to include valganciclovir on their essential drugs lists in line with the WHO recommendations, and to include CMVR screening and treatment in the package of advanced HIV care. At the same time, access to simple, affordable, effective diagnostic tools for CMV is required, which would support earlier detection and treatment of CMVR, and facilitate a reduction in the burden of under-diagnosed extra-ocular CMV disease.

## Figures and Tables

**Fig. 1:**
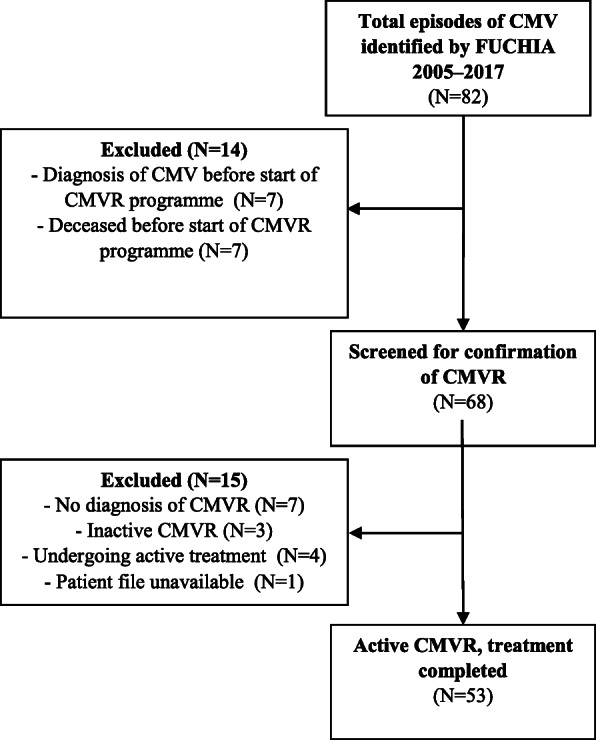
Inclusion criteria for patients in a retrospective study of a CMVR screening and treatment programme at MTY Clinic, Myanmar, 2013–2017. CMV, cytomegalovirus; CMVR, cytomegalovirus retinopathy; MTY, Mittar Yeik.

## Data Availability

The dataset generated by this study is not publically available owing to the sensitive and confidential nature of data regarding HIV positive patients in geographically specific locations.

## Appendix 1

**Table 5:** ICD-10 classification of visual impairment [19].

Category of visual impairment		Visual acuity	
		Worse than	Equal to/better than
0	Mild/none	N/A	6/18
1	Moderate	6/18	6/60
2	Severe	6/60	3/60
3	Blindness	3/60	1/60
4	Blindness	1/60	Light perception
5	Blindness	No light perception	N/A

*N/A* Not applicable

## Abbreviations

AIDS: Acquired immunodeficiency syndrome
ART: Antiretroviral therapy
CMV: Cytomegalovirus
CMVR: Cytomegalovirus retinitis
ERB: Ethics review board
HIC: High-income countries
HIV: Human immunodeficiency virus
IOG: Intra-ocular ganciclovir
ICD-10: International statistical classification of diseases and related health problems
LMIC: Low- and middle-income countries
MSF: Medecins Sans Frontieres
MTY: Mittar Yeik clinic
NGO: Non-government organization
OI: Opportunistic infection
RNA: Ribonucleic acid
VA: Visual acuity
